# The global evaluation of nursing interventions on patient outcomes in children with pneumonia: a systematic review and meta-analysis

**DOI:** 10.3389/fped.2026.1730779

**Published:** 2026-08-07

**Authors:** Lijuan Zhou, Hui Meng, Liming Cao

**Affiliations:** Department of Infectious Disease, Children's Hospital of Nanjing Medical University, Nanjing, China

**Keywords:** complications, meta-analysis, pediatrics, pneumonia, systematic review

## Abstract

**Background/objectives:**

Pediatric patients diagnosed with pneumonia may experience a reduced incidence of complications when provided with appropriate nursing care. Numerous studies have investigated the impact of interventions on outcomes in children with pneumonia. However, the findings have been contradictory, and the effectiveness of these interventions on patient outcomes can differ. Therefore, it is crucial to compile and compare the results of these studies across various nursing intervention types. We conducted a systematic review and meta-analysis to evaluate the effectiveness of different interventions on patient outcomes in pediatric pneumonia cases.

**Methods:**

A comprehensive search was conducted across five reputable databases (Scopus, PubMed, Medline, Embase, and Web of Science) using three groups of keywords up to September 2025, following PRISMA guidelines. All types of studies were included if they involved nursing interventions in children with pneumonia. For the meta-analysis, nursing satisfaction, complication rates, and their 95% confidence intervals (CIs) in case-control studies were calculated using a random-effects model. The GRADE assessment was also performed. The quality of the included studies was evaluated using the Joanna Briggs Institute (JBI) critical appraisal tools.

**Results:**

This review analyzed a total of 40 studies encompassing 166,859 cases of pediatric pneumonia, including all study designs implemented in this field. Targeted nursing care, comprehensive nursing care, and hierarchical chain nursing care were identified as the most effective interventions. However, medication programs, educational nursing interventions, and positioning care programs, while cost-effective, can significantly enhance patient outcomes. Following these interventions, nursing satisfaction rates improved significantly, with a pooled standardized mean difference (SMD) of 1.79 (95% CI: 1.33–2.25), and complication rates decreased, also with an SMD of −3.81 (95% CI: −4.17–−3.45).

**Conclusions:**

Nursing care interventions for children with pneumonia can significantly improve patient outcomes. It is crucial to develop effective and cost-efficient nursing strategies specifically tailored to pediatric pneumonia cases. The successful implementation of high-impact nursing interventions requires close collaboration among individual care providers, leadership, and policymakers. Policymakers and healthcare practitioners must carefully evaluate healthcare priorities alongside the effectiveness, benefits, and potential risks associated with these interventions.

**Systematic Review Registration:**

PROSPERO CRD420251164790.

## 1 Introduction

Pneumonia is an inflammation of the lung parenchyma. In 1991, William Osler, in the fourth edition of his book (“the principles and practice of medicine”), pointed out that pneumonia is the most deadly and, at the same time, the most widespread disease, “captain of the men of death.” Despite the passage of more than a century since then, pneumonia remains important as a clinical syndrome. It is one of the ten most common causes of death among all age groups in the United States, the sixth leading cause of death in people 50 years of age and older, and the most common cause of death from infection (1, 2).

The worldwide rate of pneumonia among children under 5 years old was 107.7 episodes per 1,000 children. By region, the incidence per 1,000 children was as follows: 107.1 cases in Central Europe, Eastern Europe, and Central Asia; 94.9 cases in Latin America and the Caribbean; 120.4 cases in Southeast Asia, Eastern Asia, and Oceania; 133.2 cases in North Africa and the Middle East; and 100.6 cases in sub-Saharan Africa (3, 4).

Nursing practice reflects the ongoing development of nursing as a profession. Pediatric nursing increasingly emphasizes professional skills, clinical judgment, and outcomes sensitive to nursing care, rather than merely task-based interventions. In nursing, competency refers to the ability to carry out a specific task or action combined with the necessary knowledge. This means that being competent involves not only executing tasks correctly but also understanding why, when, and how to act. These essential competencies serve as the basis for professional nursing practice, guiding how individual nurses effectively apply a range of skills in various settings. Contemporary nursing models consistently highlight that competence is multifaceted. The American Association of Colleges of Nursing (AACN) identifies core nursing competency areas, including knowledge for nursing practice, person-centered care, population health, nursing scholarship, quality and safety, interprofessional collaboration, system-based practice, informatics and healthcare technologies, professionalism, and personal, professional, and leadership development. These areas demonstrate that nurses function not only as caregivers but also as coordinators, educators, and advocates within healthcare systems (5, 6).

The scope of interventions implemented by the healthcare system to address pneumonia includes both vaccine-based and non-vaccine strategies. Vaccination has proven effective against several primary causative agents of pneumonia; however, its protective effects are limited to particular pathogens or specific pathogenic serotypes. Non-vaccine approaches that have demonstrated potential effectiveness include caregiver education and facilitating referrals to medical services during immunization appointments. Managing patients diagnosed with pneumonia requires an interdisciplinary framework that integrates expertise and methodologies from various academic disciplines (7, 8). Pediatric nursing is inherently family-centered. Family-centered care (FCC) is a fundamental approach in pediatric nursing that emphasizes collaboration among healthcare providers, patients, and their families. It is based on the understanding that families play a crucial role in a child's treatment and healing process (9).

**Figure 1:**
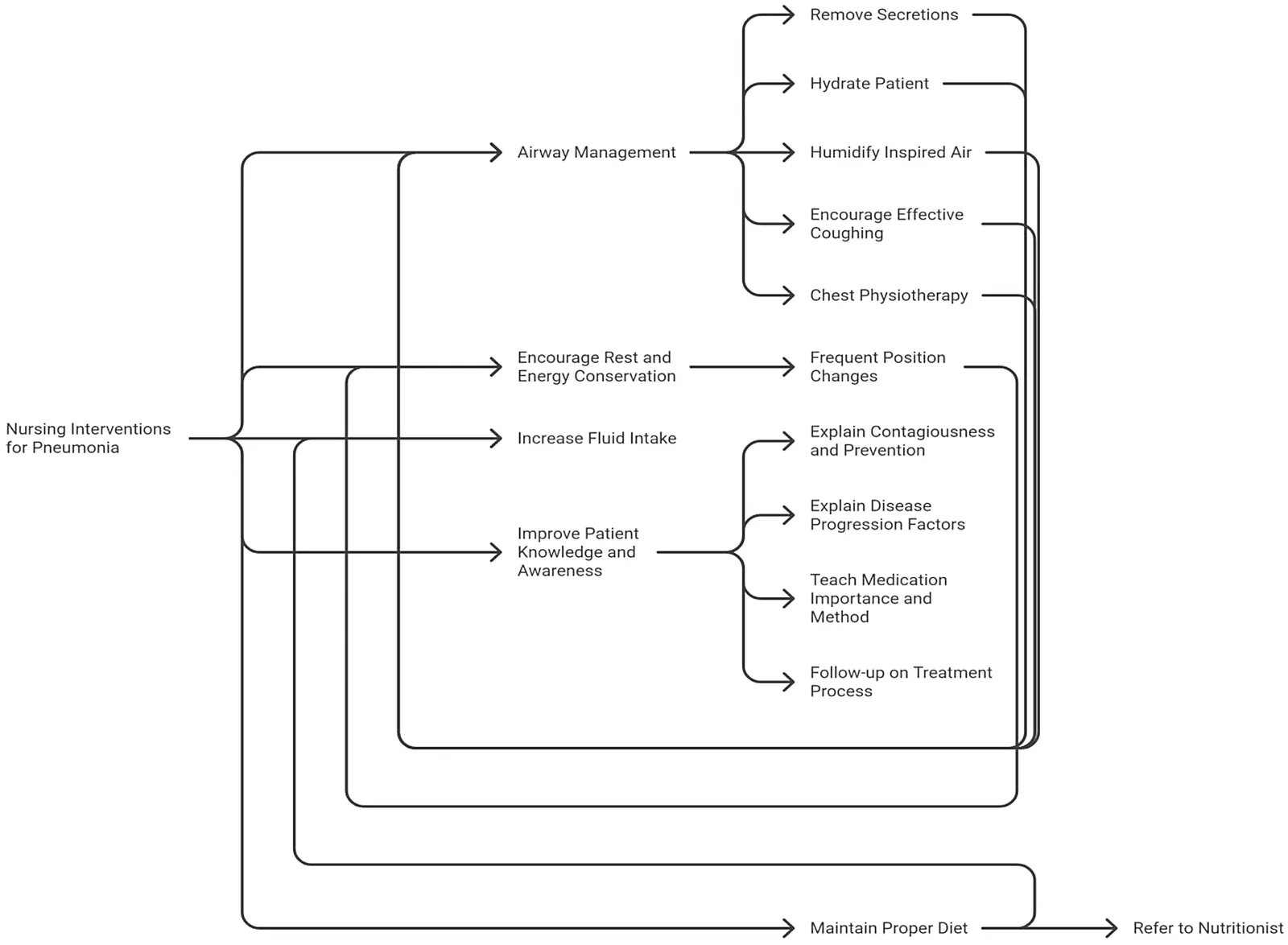
Nursing interventions for pneumonia in children.

Individuals of all age groups can exhibit symptoms such as fever, cough, chest pain, mucus production, and difficulty breathing. While many improve with outpatient treatment, pneumonia still results in a significant number of hospitalizations. Nurses play a crucial role in managing pneumonia. In primary care, they may participate in initial evaluations, triage patients based on urgency, and provide follow-up care. In hospitals, nurses assist by monitoring patients, administering treatments, supporting recovery, and working to prevent complications. Nursing interventions include promptly assessing the severity of the condition, making timely referrals to the hospital, providing oxygen and hydration support, closely monitoring for any worsening symptoms, and participating in discharge planning. Additionally, nurses play a crucial role in educating patients and their families, ensuring adherence to antibiotic treatments, and monitoring recovery progress. Figure 1 illustrates these interventions (10–14). Pediatric patients diagnosed with pneumonia may experience a reduced incidence of complications when provided with appropriate nursing care. Numerous studies have investigated the impact of interventions on outcomes in children with pneumonia (10–12). However, the findings have been contradictory, and the effectiveness of these interventions on patient outcomes varies. Therefore, conducting a systematic review in this field is necessary (15, 16).

Several reviews have been conducted to investigate nursing interventions on patient outcomes in pediatrics with various diseases, such as bronchial asthma (17) and type 1 diabetes (18). However, no systematic review has been carried out to evaluate the effects of nursing interventions on patient outcomes in pediatric pneumonia. Therefore, the general aim of this systematic review and meta-analysis is to assess the impact of nursing competencies on outcomes in children with pneumonia. The specific objectives of the study are: 1) to identify which interventions are most effective; 2) to evaluate the effectiveness of these interventions in improving nursing satisfaction rates; and 3) to assess their effectiveness in reducing complication rates. The results of this study will provide valuable insights into the effectiveness and magnitude of these interventions, as well as their implications for clinical practice and future research for healthcare professionals and researchers. Additionally, the findings will inform the development of interventions aimed at improving outcomes in pediatric pneumonia cases.

## 2 Methods

### 2.1 Protocol

**Figure 2:**
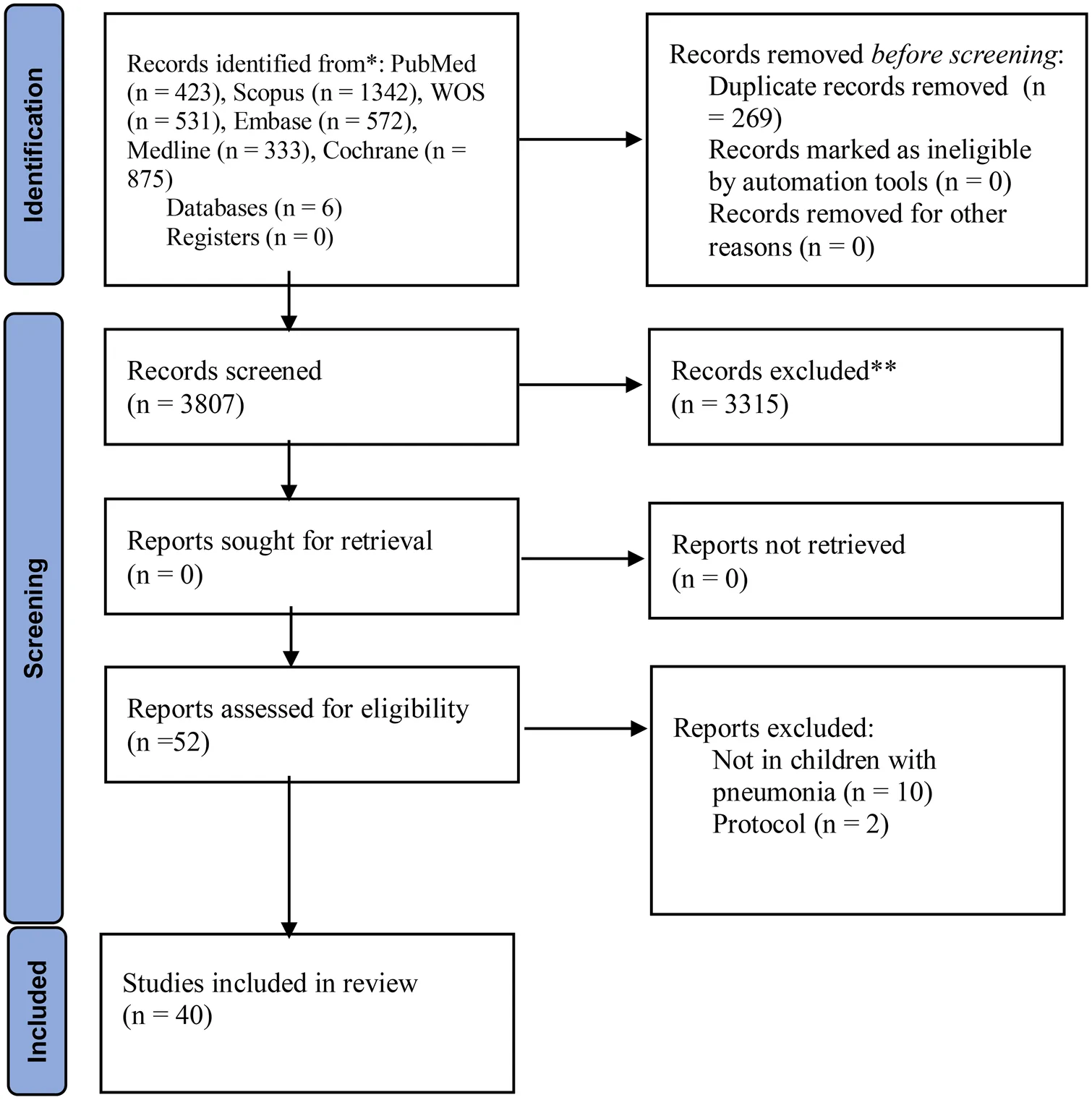
PRISMA flowchart.

This study was conducted following the PRISMA guidelines for systematic reviews and meta-analyses (see Figure 2). PRISMA checklist is provided in the Supplementary Material 1. The research protocol was previously registered in the International Prospective Register of Systematic Reviews (PROSPERO) under the number CRD420251164790.

### 2.2 PICO model

The PICO model was used to clarify the research question, and studies that did not meet the criteria established by this model were excluded (19). The criteria applied in the present study are outlined below.

#### 2.2.1 Types of population

In the current study, we included pediatric pneumonia cases. All patients included in this review were under 18 years old.

#### 2.2.2 Types of intervention

We included studies that implemented any nursing intervention in cases of pediatric pneumonia.

#### 2.2.3 Types of comparator

In this context, interventions were compared either between the routine care group and the intervention group or by evaluating conditions before and after the intervention.

#### 2.2.4 Types of outcome(s)

The study examined the effectiveness of interventions on patient outcomes, including complication rates, quality of life, pulmonary function, clinical symptoms and signs, serum biomarkers, and length of hospitalization in cases of pediatric pneumonia. It also evaluated nursing-sensitive outcomes such as nursing satisfaction and therapeutic efficacy.

### 2.3 Literature searches

A thorough literature search was conducted during the first week of September 2025 using the databases Scopus, PubMed, Medline, Embase, and Web of Science. These databases were chosen because they broadly cover peer-reviewed published papers. Additionally, a previous search was conducted on the terms and keywords of published articles related to this topic. To ensure a comprehensive search, three groups of keywords were combined using the search operator “AND.” The first group of keywords was Nurs*. The second group included child* OR pediatric. The third group consisted of pneumon* OR “lung inflammation” OR “pulmonary inflammation” Additionally, the references of the papers included in this review, as well as other relevant systematic reviews, were examined to identify further related studies. A library expert approved review process. Full database-specific search strings are not provided in Supplementary Material 2.

### 2.4 Selection criteria

This review offers a comprehensive assessment of various study designs, including case-control, cross-sectional, cohort, and case report studies. All study designs were eligible for inclusion; however, no randomized controlled trials (RCTs) have been conducted in this field. The search was unrestricted by time or location, and only articles published in English were included. Inclusion criteria required studies to focus on pediatric patients hospitalized with pneumonia who had undergone an intervention and to be published in peer-reviewed, English-language journals. Studies were excluded if the full text was inaccessible despite attempts to contact the authors. Additionally, conference papers or proceedings, book chapters or books, short surveys, notes, editorials, and letters to the editor were also excluded, because they do not undergo the same blind evaluation and critique process as peer-reviewed journal articles.

### 2.5 Study selection

Two researchers independently reviewed the titles and abstracts of all articles to exclude those deemed irrelevant by both reviewers. Based on predefined eligibility criteria, full-text versions of potentially relevant papers were obtained and assessed for inclusion by the two researchers. The investigators reached consensus on all studies, making the involvement of a third researcher — initially planned to resolve uncertainties or disagreements—unnecessary.

### 2.6 Methodological assessments

The quality of the selected studies was assessed using the Joanna Briggs Institute (JBI) Critical Appraisal Tools. The critical appraisal included assessing several elements of the studies: the suitability of the sample frame, the selection process of study participants, whether the sample size was sufficient, comprehensive descriptions of the study subjects and their environment, the data analysis procedures, the validity of the methods used, the reliability of standard measurements, the appropriateness of the statistical analyses, and whether the response rate was adequate (20). The total number of positive responses to the criteria was calculated, and these scores were used to classify the studies into three categories: low (Q1), moderate (Q2), and high quality (Q3). This score did not influence the inclusion or exclusion of papers from the review; all included studies were categorized based on these criteria. In this review, two researchers (LZ, HM) independently evaluated the quality of the papers using the appraisal checklist.

### 2.7 Data extraction

Two reviewers examined all full-text articles (LZ, HM) and extracted data according to predefined criteria. The relevant information was gathered from the selected studies and organized by the same two reviewers, including details such as the first author, publication year, country, number of participants, participants' age, pneumonia type, type and duration of nursing intervention, outcome measures, service providers, and outcomes (e.g., satisfaction rate, complication rate).

### 2.8 Data synthesis and analysis

Cohen's kappa coefficient was used to assess the agreement between investigators during data extraction (21). The kappa values obtained for the initial and subsequent stages were 0.94 and 0.96, respectively, indicating strong agreement between the two investigators. This study utilized both qualitative and quantitative approaches. The qualitative analysis described characteristics of the included studies, such as study populations, countries, interventions, and quality. The quantitative analysis reported nursing satisfaction, complication rates, and 95% confidence intervals (CIs) for effect sizes. The meta-analysis was conducted on case-control studies. To account for differences in measurement scales used to assess nursing satisfaction or complication rates across studies, the standardized mean difference (SMD) between case and control groups was calculated for each study. Heterogeneity among the selected studies was evaluated using the Cochrane Q test and the I² statistic. Because the *p*-value for heterogeneity was less than 0.1, a random-effects model was employed (22). Subgroup analyses were performed according to geographical location, income level, and study year to identify principal sources of heterogeneity. For these analyses, countries were classified into low- and middle-income (LMIC) and high-income (HIC) groups based on the World Bank's criteria (23). Furthermore, the studies were stratified into four geographic regions: Europe, the Middle East, East/Southeast Asia, and the Americas. Temporal categorization was also applied, distinguishing studies conducted in or before 2020 from those conducted after 2020. To assess whether heterogeneity was significantly reduced within these predefined subgroups (income level, region, and study period), the Q-test and I² statistic were utilized. Additionally, funnel plots were generated to evaluate potential publication bias, and the Egger regression test was employed to detect any asymmetry indicative of such bias (24). The certainty of the evidence was evaluated based on factors such as random sequence generation and allocation concealment, blinding bias involving participants and researchers (including allocation concealment to study groups, intention and methods of blinding, and effectiveness of blinding), blinding bias related to outcome assessors (whether reported, requiring researcher judgment, or not), attrition bias (due to incomplete data or exclusion from analysis), and reporting bias (selective reporting of outcomes). Each factor was classified as low risk, high risk, uncertain risk, or not applicable. A pilot test for assessing bias risk was performed on a sample of studies. The risk of bias was taken into account when determining the overall certainty of the evidence using the GRADE (Grading of Recommendations Assessment, Development and Evaluation) approach (25). Data analysis was conducted using STATA version 14.2.

## 3 Results

### 3.1 Study characteristics

This review analyzed 40 studies involving a total of 166,859 pediatric pneumonia cases. The initial search identified 4,076 records. After duplicates were removed, 3,807 records were screened based on their titles and abstracts for relevance. Among these, 3,755 were deemed irrelevant and excluded, leaving 52 full-text articles for further evaluation. Ultimately, 12 articles were excluded, resulting in 40 articles being included in the current review (Figure 2).

The study details are summarized in Supplementary Table S1. The research was conducted across various cultural contexts: 35 studies took place in Asia, including 30 in China, 2 in Indonesia, 1 in Thailand, 1 in Bangladesh, and 1 in Turkey. Five studies were conducted in Africa, with one each in Kenya, Zimbabwe, South Africa, Ethiopia, and Egypt. The study designs also varied: 32 were case-control, 5 were cross-sectional, 2 were care-report, and 1 was a cohort study. Additionally, the majority of studies were published in 2025 (12 papers; 30.0%), 2024 (5 papers; 12.5%), and 2023 (5 papers; 12.5%), highlighting the global importance of the topic. Following a qualitative assessment of the included papers, 15 were rated as high quality (Q1), 10 as moderate quality (Q2), and 15 as low quality (Q3).

### 3.2 Characteristics of the interventions

The duration of nursing interventions ranged from 5 days to 8 months. The studies reported positive outcomes for children with pneumonia who participated in various intervention programs. Targeted nursing care, comprehensive nursing care, and hierarchical chain nursing care were identified as the most effective interventions, as patient outcomes improved significantly following their implementation. However, medication programs, educational nursing interventions, and positioning care programs, while cost-effective, can significantly enhance patient outcomes. Detailed descriptions of the interventions are provided in Table 1.

**Table 1:** Findings of subgroup analysis.

Subgroup analysis					
Subgroup	Category (number of studies)	Pooled complication rate (%) [95% CI]	I2 (%)	Q statistic (df)	*p* of heterogeneity
Income level	High income (-) LMICs (7)	- −3.45 [−3.82, −3.08]	- 99.4	- 6	- < 0.001
Region	Europe (-) East/southeast Asia/Oceania (7) Middle East (-) Americas (-)	- −3.45 [−3.82, −3.08] - -	- 99.4 - -	- 6 - -	- < 0.001 - -
Study date	In or before 2020 (2) After 2020 (6)	−2.27 [−3.51, −1.03] −3.03 [−3.41, −2.66]	85.8 99.4	1 5	0.008 < 0.001

### 3.3 Findings of the meta-analysis

**Figure 3:**
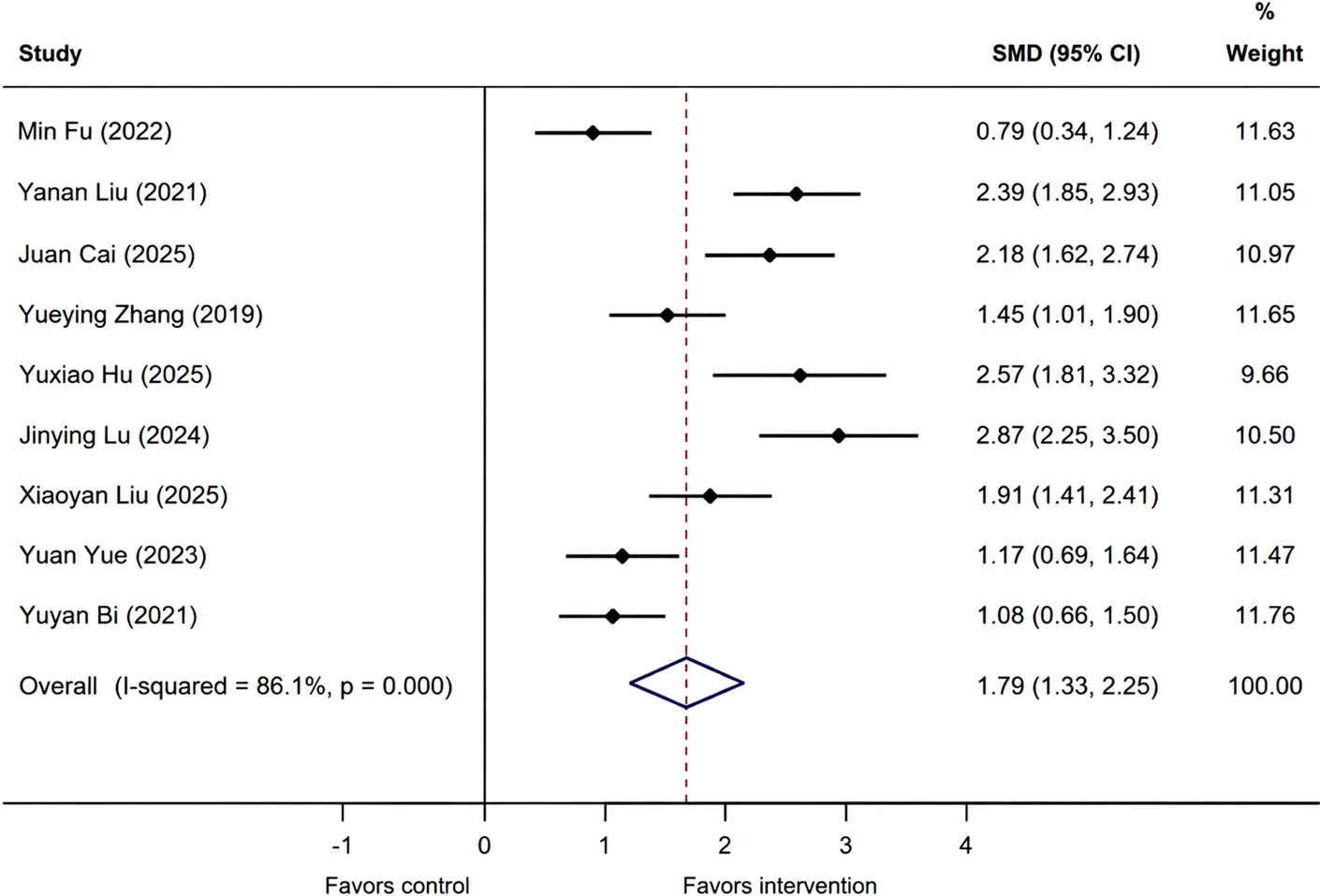
Pooled SMD for nursing satisfaction rates.

**Figure 4:**
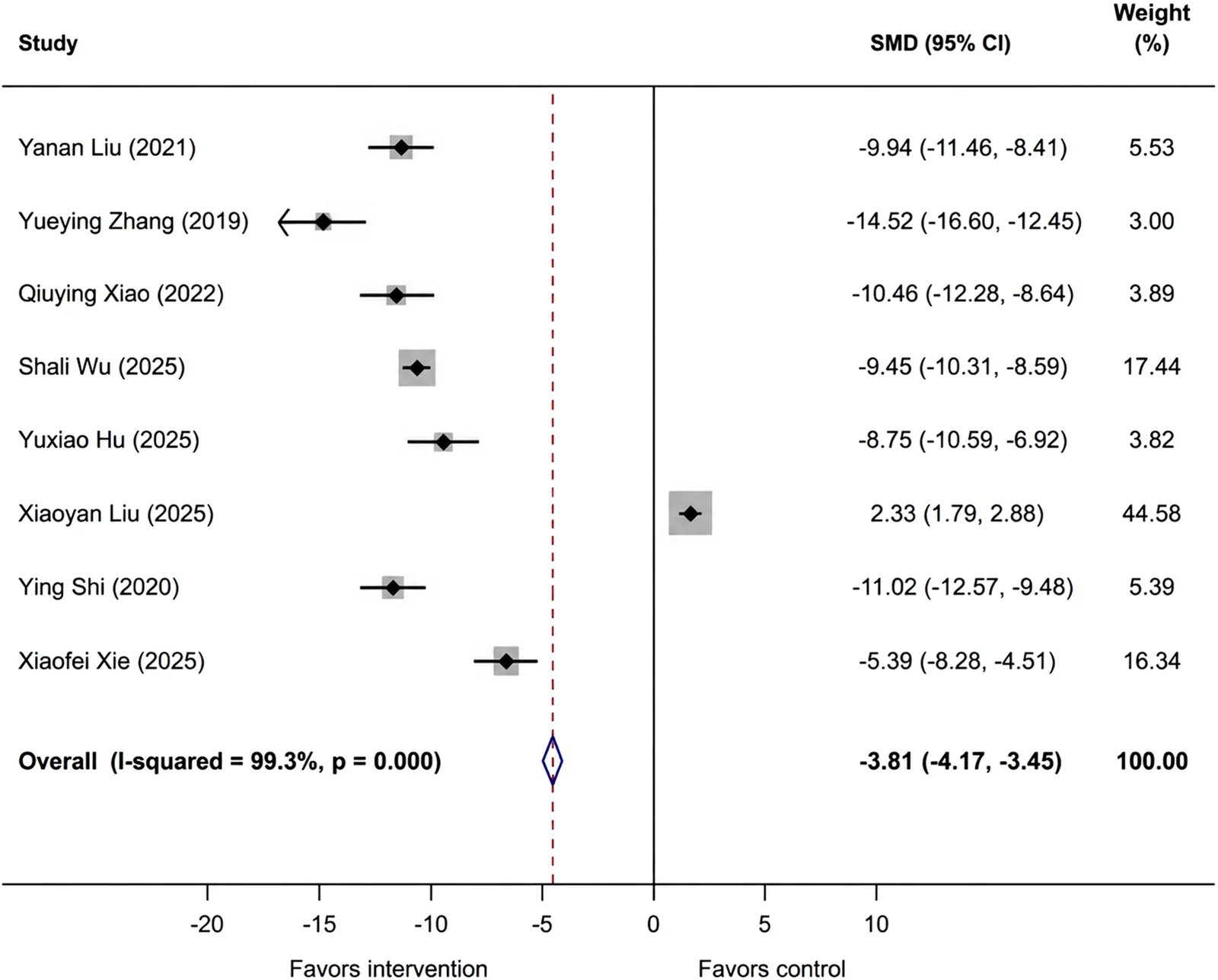
Pooled SMD for complication rates.

**Figure 5:**
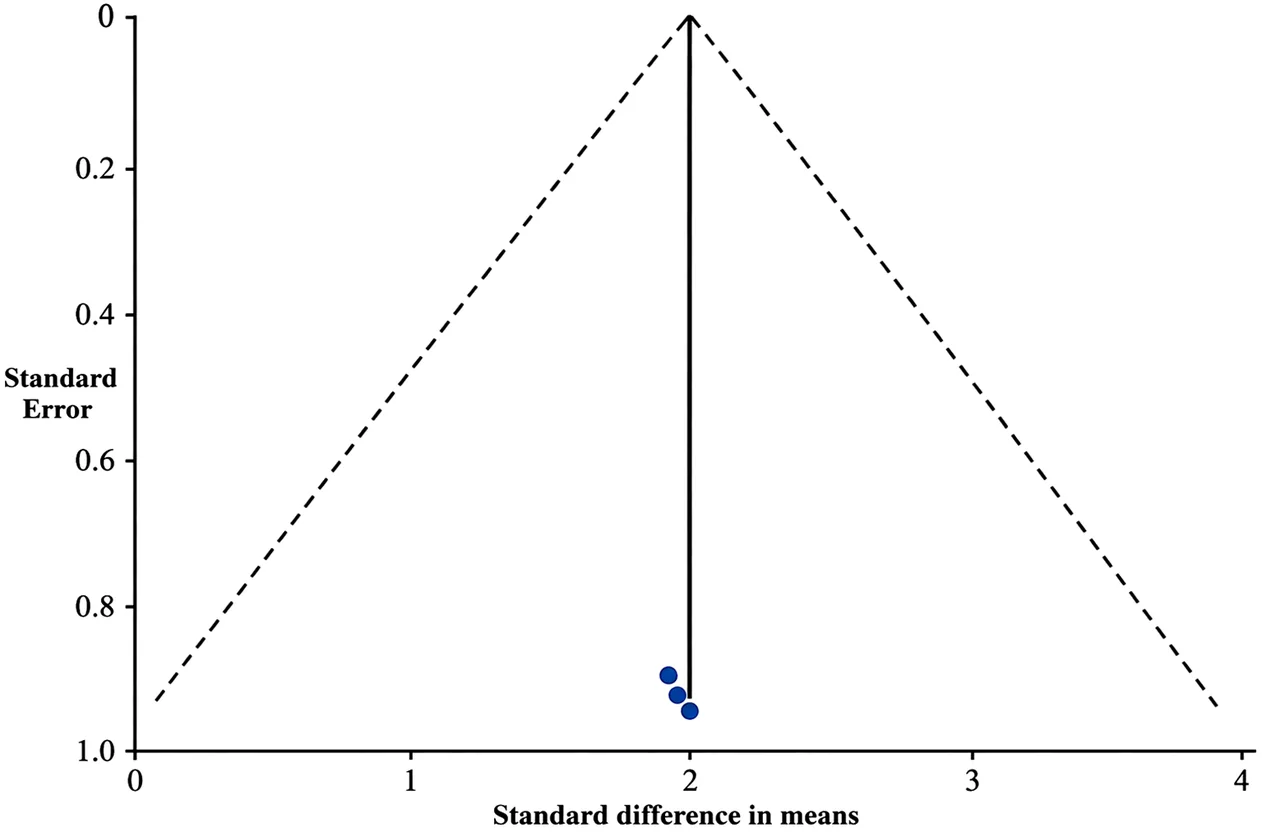
Funnel plot to examine possibility of publication bias.

A total of 13 case-control studies reporting nursing satisfaction or complication rates were included in the meta-analysis. Following the interventions, nursing satisfaction rates showed a significant increase, with a combined standardized mean difference (SMD) of 1.79 (95% CI: 1.33–2.25), and complication rates decreased, also with an SMD of −3.81 (95% CI: −4.17–−3.45). The overall pooled SMD results for nursing satisfaction and complication rates are illustrated in Figures 3, 4, respectively. Table 1 summarizes the findings of the subgroup analysis. Publication bias was detected in the studies, as indicated by Egger's test (*P* < 0.05) (Figure 5). Following the GRADE methodology criteria, the overall certainty of scientific evidence was assessed as low quality. The findings of the meta-analysis in this study should be interpreted with great caution due to significant heterogeneity, quality concerns, and absence of RCT studies.

## 4 Discussion

The current review aimed to evaluate the evidence regarding the efficacy various interventions on patient outcomes in pediatric pneumonia cases. Nearly all 40 studies included in this review reported favorable patient outcomes following the implementation of different intervention types. Pooled estimates from thirteen studies demonstrated increased nursing satisfaction and reduced complication rates in pediatric pneumonia cases after the interventions. The findings of the meta-analysis in this study should be interpreted with great caution due to significant heterogeneity, quality concerns, and absence of RCT studies.

The results of the study showed that nursing interventions effectively promote faster recovery, reduce adverse reactions, improve overall nursing satisfaction in children with pneumonia, and significantly increase treatment outcomes (35). The study emphasized the role of multidisciplinary care teams in supporting the management of childhood pneumonia. While primarily focusing on behavioral interventions, it highlights the importance of family involvement and appropriate approaches to meet individual needs (46). Effective interventions include skills education programs and family interventions, which can increase adherence to care regimens. Additionally, integrating behavioral health into children's routine care is critical for promoting meaningful improvements in their well-being (59).

Based on the study's findings, it is recommended that the educational needs of children and their mothers be assessed upon admission. Appropriate educational content should then be developed and delivered to help reduce mothers' uncertainty about the illness. Elevated levels of stress and anxiety, negative changes in self-care, social, and work life, as well as the use of ineffective coping strategies, increase the risk of burnout and depression. Conversely, employing effective stress-coping strategies reduces this risk. Additionally, the duration of the child's illness and family income are significant factors influencing the risk of depression, while increased parental perceived competence in managing the illness serves as a protective factor against burnout (49, 60).

The study results demonstrated that telephone case management, coping skills training, motivational interviewing, home visit training programs, and nursing interventions significantly reduced disease incidence. Systemic nursing interventions encompass coherent routines, effective care methods, a unified set of goals, flexibility and adaptability to potential challenges and crises, willingness to share time, effective communication patterns, support and encouragement mechanisms, and a clear understanding of the child's and family's illness situations. These factors should be considered to improve health outcomes (10, 59).

Care for children and their families is increasingly focused on disease control, often at the expense of expectations, needs, and psychosocial outcomes. Therefore, both the child and family should actively participate in collaboratively building a support network to develop strategies for coping with the intense and ongoing challenges of daily life. Nursing interventions across various health disciplines should also be considered, as this approach can facilitate integrated and compassionate care for children and their families. To help children and families manage the psychological effects of illness, build resilience, and adapt to chronic conditions, nurses must understand the child's behavior and social integration. This understanding enables nurses to design interventions that address the specific challenges the child faces. Nurses play a critical and multifaceted role caring for children with pneumonia and their families. These roles include providing direct clinical care, education, psychosocial support, interdisciplinary coordination, and involvement in treatment decisions. Because pneumonia is a chronic disease requiring daily management, the role of nurses extends beyond medical treatment to empowering the child and family to independently manage the condition (12, 61).

In the current review, various interventional approaches were employed, which targeted nursing care, comprehensive nursing care, and hierarchical chain nursing care were identified as the most effective interventions.

Targeted nursing is a well-established nursing approach that offers specific care measures tailored to patients. This type of nursing intervention is a key form of comprehensive care that addresses both the physical and psychological needs of patients through customized intervention plans based on an accurate evaluation of their clinical condition, aiming to enhance their quality of life. Targeted nursing has been extensively applied in the care of children with pneumonia. Hua et al. conducted a study to investigate the effects of targeted nursing care combined with Qingfei Huayu decoction (traditional medicine approach) on outcomes in children with mycoplasma pneumoniae pneumonia. Traditional medicine emphasizes natural treatments and a holistic, individualized approach to restoring harmony among the mind, body, and surroundings. The findings showed that the intervention can considerably increase clinical efficacy, advance symptom relief, and enhance the body's inflammatory status (27).

Comprehensive nursing is a patient-centered approach in healthcare that focuses on personalized care. It assesses and evaluates both the physical and psychological aspects of patients from multiple perspectives and levels, aiming to promote their recovery and improvement (46, 50). Through this method, nurses develop their ability to identify and understand the connections between a patient's physical health and their psychological and social difficulties. Therefore, since this type of nursing intervention addresses various facets of the patient, it is anticipated to have the most significant impact on the patient's progress (46, 50). Liu et al. conducted a study to investigate the effects of comprehensive nursing based on the 3H model on outcomes in children with severe pneumonia. The intervention includes monitoring of vital signs, respiratory care, psychological care, ward optimization, hotel-style (hotel) service, hospital-style (hospital) service, and home-style (home) service. The findings demonstrated that the intervention was significantly effective in managing pneumonia in the patients (length of hospital stay (mean): case = 8.56, control = 15.16; FEV1 (L): case = 3.54, control = 2.81; FVC (L): case = 2.34, control = 1.76; MVV (L): case = 59.47, control = 51.75; PEF (L/min): case = 198.64, control = 178.32; FEV1/FVC: case = 80.57, control = 72.71; nursing satisfaction (%): case = 97.78, control = 82.22; compliance rate (%): case = 75.56, control = 93.33) (46).

In healthcare settings, the hierarchical chain nursing plays a vital role in influencing how patient care is provided and shaping the overall work environment. However, if the hierarchy is poorly managed or harmful, it can compromise patient safety and negatively impact staff morale. The hierarchical chain nursing encompasses its organizational structure and the importance it carries within the healthcare context. It impacts the staff's interactions and teamwork, ultimately affecting patient outcomes. Healthcare leaders can obtain important insights into comprehending and enhancing the hierarchical chain nursing to create a safer and more supportive environment. Grasping the structure of the hierarchical chain nursing is crucial for effective leadership and efficient resource management within healthcare organizations (13, 45). Wang et al. conducted a study to investigate the effects of Hierarchical chain nursing model combined with Feilike mixture treatment on outcomes in children with pneumonia. The findings showed that the intervention effectively enhanced the therapeutic efficacy [total effective rate (%): case = 97.5, control = 77.5] (13).

Various outcomes have been used in other studies to assess effectiveness of the interventions, such as acute physiology and chronic health evaluation II (APACHE II) scoring system (62), St. George's respiratory problems questionnaire (SGRQ) score (63), and coagulation function (63).

The findings of this review showed that several studies examined the effects of educational nursing programs and other interventions, including medication management and positioning care programs, on children with pneumonia. Interestingly, these interventions, while cost-effective, can significantly enhance patient outcomes. However, their impact was somewhat less significant compared to other nursing interventions.

Parvez et al. conducted a study to investigate the effects of educational nursing program on outcomes in children with pneumonia. The findings showed that the intervention improved knowledge and behavior of the patients' mothers [knowledge (mean): case = 34.64, control = 25.04; behavior (mean): case = 17.68, control = 6.64] (34). Keeley et al. conducted a study to examine the effects of medication program (group A: sulfamethoxazole + trimethoprim versus group B: procaine penicillin) on outcomes in children with pneumonia. The results showed that both interventions were highly and equally effective [referred to hospital (%): group A = 12.0, group B = 10.0] (36).

Finally, these nursing interventions support the Sustainable Development Goals (SDGs) by tackling health, social, and environmental factors through clinical care, education, and advocacy. Nurses contribute to SDG 3 (good health and well-being) by delivering preventive services, managing pneumonia in children, and encouraging healthy habits among affected children. They also advance gender equality (SDG 5), enhance health equity (SDG 10), and encourage sustainable healthcare practices, serving as important leaders in community health and environmental care.

### 4.1 Implication for nursing practice

Pneumonia is a growing epidemic worldwide. Nursing interventions play a crucial role in improving patients' conditions. Family-centered education and empowerment, psychological and emotional support, continuous follow-up and care, interdisciplinary communication and service coordination, and the use of technology and digital education are key elements that nursing staff must consider when caring for children. Nurses play a vital role in preventing long-term complications of the disease by promoting healthy lifestyles, enhancing coping skills within the family, and raising awareness about secondary risk factors such as obesity or and physical inactivity.

Overall, nurses, by fulfilling a variety of professional roles, play a fundamental part in improving the health outcomes of sick children, enhancing the quality of life for families, and promoting the efficiency of the healthcare system.

Investment in specialized training for nurses, particularly in the field of pediatric pneumonia, can help establish a responsive and sustainable healthcare system.

### 4.2 Limitations

First, none of the included articles is a randomized controlled trial (RCT), which is essential for evaluating the effectiveness of interventions. Second, the single-center nature of the study restricts the applicability of the findings to other healthcare settings or patient groups. It remains unclear if similar results would be seen in different clinical contexts or regions with varying healthcare resources. Third, the relatively brief follow-up, which concluded at hospital discharge, limited the ability to evaluate long-term outcomes and possible delayed complications, especially in children with pneumonia. Fourth, the study did not consider several potential confounding factors, including disease severity, existing comorbidities, and differences in treatment approaches. These unmeasured factors may have affected the results, making it challenging to attribute the observed benefits solely to the nursing care provided. To overcome these limitations, future studies should be randomized controlled trials, multicenter in design, include extended follow-up periods, and rigorously control for confounding variables. This approach will help confirm and strengthen the reliability of the findings.

## 5 Conclusion

The findings of this review indicate that various nursing interventions can effectively improve both the physical and psychological well-being of children with pneumonia. Targeted nursing care, comprehensive nursing care, and hierarchical chain nursing care showed the greatest effectiveness. Meanwhile, medication programs, educational nursing interventions, and positioning care programs, although more affordable, also contribute positively to patient recovery. Therefore, these nursing interventions can be selected and implemented based on the desired level of effectiveness and specific objectives. It is recommended that future research develop a high-impact nursing intervention approach that balances efficacy and cost. The successful implementation of high-impact nursing interventions requires close collaboration among individual care providers, leadership, and policymakers. It is essential for policymakers and healthcare practitioners to carefully evaluate healthcare priorities alongside the effectiveness, advantages, and potential risks associated with these interventions. The findings of the meta-analysis in this study should be interpreted with great caution due to significant heterogeneity, quality concerns, and absence of RCT studies.

## Data Availability

The original contributions presented in the study are included in the article/Supplementary Material, further inquiries can be directed to the corresponding authors.
