# Supplementary material for: The global evaluation of nursing interventions on patient outcomes in children with pneumonia: a systematic review and meta-analysis
Source: Front Pediatr. 2026 Aug 7;14:1730779. doi: 10.3389/fped.2026.1730779 (PMC13493380; doi:10.3389/fped.2026.1730779)
Supplement: Supplementary file 1 [file Supplementaryfile1.docx]

**Embase**

nurs*:ab,ti AND (child*:ab,ti OR pediatric:ab,ti) AND (pneumon*:ab,ti OR 'lung inflammation':ab,ti OR 'pulmonary inflammation':ab,ti) AND [embase]/lim

**Medline**

nurs*:ab,ti AND (child*:ab,ti OR pediatric:ab,ti) AND (pneumon*:ab,ti OR 'lung inflammation':ab,ti OR 'pulmonary inflammation':ab,ti) AND [medline]/lim

**PubMed**

((Nurs*[Title/Abstract]) AND ((child*[Title/Abstract]) OR (pediatric[Title/Abstract]))) AND (((pneumon*[Title/Abstract]) OR ("Pulmonary Inflammation"[Title/Abstract])) OR ("Lung Inflammation"[Title/Abstract]))

**WOS**

## TS=(Nurs*)

## (TS=(child*)) OR TS=(pediatric )

## ((TS=(pneumon*)) OR TS=(“Pulmonary Inflammation”)) OR TS=(“Lung Inflammation”)

## Scopus

## ( ( TITLE-ABS-KEY (Nurs*) ) AND ( TITLE-ABS-KEY (child*) OR TITLE-ABS-KEY (pediatric ) ) ) AND ( ( TITLE-ABS-KEY (pneumon* ) OR TITLE-ABS-KEY (“Pulmonary Inflammation”) OR TITLE-ABS-KEY (“Lung Inflammation”) ) )
