# Supplementary material for: The global evaluation of nursing interventions on patient outcomes in children with pneumonia: a systematic review and meta-analysis
Source: Front Pediatr. 2026 Aug 7;14:1730779. doi: 10.3389/fped.2026.1730779 (PMC13493380; doi:10.3389/fped.2026.1730779)
Supplement: Supplementary file 2 [file Table1.docx]

**Table S1** The total information of the extracted articles.

| Author (year) | Country | Study type | Sample size | Gender | Age | Type of pneumonia | Nursing intervention type | Nursing intervention duration | Outcome measures | Service providers | Outcome (OR, satisfaction rate, complication rate or etc.) | Quality |
| --- | --- | --- | --- | --- | --- | --- | --- | --- | --- | --- | --- | --- |
| Min Fu (2022) (1) | China | Case-control | 83 (Case= 40, control= 43) | Male-female | Case= 13.0, control= 14.0 | Bronchopneumonia | Comfortable nursing combined with video education (nutrition care, medication care, emergency interventions, preparation of the patient, preparation of medical room, hydrotherapy, video education) | - | Researcher-made assessment criteria | Nursing staff | The intervention improved health level and degree of confidence (nursing satisfaction (%): case= 90.2, control= 84.0) | Q2 |
| Meng Du (2025) (2) | China | Case-control | 100 (Case= 50, control= 50) | Male-female | Case= 4.40, control= 4.24 | Severe pneumonia | Evidence-based nursing program (blood monitoring, fluid management  regimens, medication care, health education) | - | Biochemical analysis, nursing satisfaction questionnaire | Nursing staff | The intervention improved treatment outcomes and patient nursing satisfaction (satisfaction (mean score): case= 89.2, control= 80.2) | Q1 |
| Yanan Liu (2021) (3) | China | Case-control | 90 (Case= 45, control= 45) | Male-female | Case= 5.72, control= 5.46 | Pneumonia | Continuous nursing program (health education, dietary guidance, respiratory tract care, psychological guidance, Discharge nursing guidance, Discharge sleep guidance, Targeted telephone follow-up, ) |  | X-ray examination, symptom checklist (SCL-90), nursing satisfaction questionnaire | Nurse | The intervention reduced the complication rate and improved patient satisfaction (nursing effectiveness (%): case= 95.55, control= 75.55; complication rate (%): case= 8.89, control= 26.67; nursing satisfaction (%): case= 91.11, control= 73.33) | Q2 |
| Fu Sl (2019) (4) | China | Case-control | 96 (Case= 48, control= 48) | - | - | Pneumonia | High quality nursing program (respiratory tract care, medication care, psychology care, diet care) | - | Nursing satisfaction questionnaire | Nursing staff | The intervention can promote the recovery of patients | Q2 |
| Zheng Hua (2024) (5) | China | Case-control | 120 (Case= 60, control= 60) | - | - | Mycoplasma Pneumoniae Pneumonia | Targeted nursing care combined with Qingfei Huayu decoction (QFHYD) | - | - | Nursing staff | The intervention can considerably increase clinical efficacy, advance symptom relief, and enhance the body's inflammatory status | Q3 |
| Hongmin Wang (2018) (6) | China | Case-control | 200 (Case= 100, control= 100) | - | - | Pneumonia | Targeted nursing care program | - | - | Nursing staff | The intervention enhanced overall treatment efficacy | Q3 |
| Meiling Wang (2023) (7) | China | Case-control | 80 (Case= 40, control= 40) | - | - | Pneumonia | Hierarchical chain nursing model combined with Feilike mixture treatment | - | Immunoblotting | Nursing staff | The intervention effectively enhanced the therapeutic efficacy (total effective rate (%): case= 97.5, control= 77.5) | Q3 |
| Novi Enis Rosuliana (2025) (8) | Indonesia | Case-control | 30 (Case= 15, control= 15) | Male-female | 1-60 months | Pneumonia | Continuous nursing care program | - | Perceived disease  severity scale, childcare stress questionnaire, child quality of life questionnaire (PedsQL), satisfaction questionnaire | Nursing staff | The intervention can promote the quality of life of patients (quality of life (%): case= 66,7, control= 26,7) | Q3 |
| Juan Cai (2025) (9) | China | Case-control | 80 (Case= 40, control= 40) | Male-female | Case= 7.75, control= 7.13 | Mycoplasma lobar pneumonia | Targeted nursing care program combined with bronchoscopy (oxygen  nebulization inhalation care, education, monitoring of the children's vital signs, providing quiet and warm environment, playing music, reading book) | 2 weeks | X-ray examination, biochemical analysis, nursing satisfaction questionnaire | Nursing staff | The intervention can effectively promote pulmonary function, increase serum interlukin-6 and procalcitonin levels, and enhance nursing satisfaction (total effective rate (%): case= 95.0, control= 77.5; nursing satisfaction (%): case= 97.5, control= 80.0) | Q3 |
| Mengqiong Zhang (2025) (10) | China | Case-control | 106 (Case= 53, control= 53) | Male-female | Case= 7.81, control= 7.68 | Mycoplasma pneumonia | Scenario‑based breathing training combined with antibiotics (video-based education: breathing exercise routines, blowing balloon exercise, string  exercise, pillow-hugging cough exercise; scenario simulation training) | 2 weeks | Pulmonary function testing instrument, biochemical analysis | Nurse, physician | The intervention can enhance lung function, reduce clinical symptoms and signs, and  decrease serum inflammatory levels (cough disappearance (mean): case= 4.62, control= 5.88; wheezing disappearance (mean): case= 5.83, control= 6.46; lung rales disappearance (mean): case= 4.67, control= 5.33; Fever resolution (mean): case= 3.56, control= 4.87) | Q1 |
| Danxia Huang (2023) (11) | China | Case report | 1 | Female | 10 | Severe interstitial pneumonia | Nursing care program (monitoring, management of immunosuppressants, control of infection, management of dynamic body fluid, personalized nutritional support, psychological care, rehabilitation exercise) | 21 days | - | Nurse | The intervention improved recovery | Q2 |
| Maricianah Onono (2018) (12) | Kenya | Cross-sectional | 161290 | Male-female | 6 months | Lower chest Indrawing pneumonia | Community case management combined with oral amoxicillin (classification of children, application of oral amoxicillin, home visit) | 2 weeks | Self-report assessment, color-coded mid upper arm circumference (MUAC) strip | Community health workers, nurse | The intervention can correctly manage the disease (treatment efficacy= 92.3%) | Q1 |
| Mohammed Masud Parvez (2010) (13) | Thailand | Case-control | 50 (Case= 25, control= 25) | Male-female | Case= 5.30, control= 3.79 | Pneumonia | Educational program | - | Structured knowledge questionnaire, behavioral checklist | Nurse | The intervention improved knowledge and behavior of the patients’ mothers (knowledge (mean): case= 34.64, control= 25.04; behavior (mean): case= 17.68, control= 6.64) | Q3 |
| Jing Chen (2025) (14) | China | Case-control | 112 (Case= 56, control= 56) | Male-female | Case= 4.47, control= 4.68 | Mycoplasma pneumonia | PDCA cyclic care  Model (education, respiratory care, nutritional care, implementing segregation measures comprising wearing masks and gloves and supplying separate sanitary facilities, complication prevention, reviewing the plan weekly) | - | Blood gas analysis, biochemical analysis, self-designed satisfaction questionnaire | Nursing staff | The intervention significantly promotes faster recovery, reduces adverse reactions, and improves overall nursing satisfaction (hospitalization time (mean): case= 7.65, control= 8.31; FVC (%): case= 91.52, control= 84.95; FEV1 (%): case= 93.52, control= 87.96; PEF (%): case= 89.61, control= 81.25) | Q2 |
| D J Keeley (1990) (15) | Zimbabwe | Follow-up | 614 (group A: sulfamethoxazole + trimethoprim= 303, group B: procaine penicillin= 311) | Male-female | 3 months - 12 years | Pneumonia | Medication program (sulfamethoxazole + trimethoprim  versus procaine penicillin) | - | X-ray examination | Nurse, physician | Both interventions were highly and equally effective (referred to hospital (%): group A= 12.0, group B= 10.0) | Q1 |
| Fang Yang (2015) (16) | China | Case-control | 90 (Case= 60, control= 30) | - | - | Severe bronchial pneumonia | Holistic nursing combined with oxygen-driving and atomized Mucosolvan inhalation | - | - | Nursing staff | The intervention was highly effective | Q3 |
| Yueying Zhang (2019) (17) | China | Case-control | 100 (Case= 55, control= 45) | - | - | Pneumonia | Risk preventive nursing program | 8 months | Blood gas analysis, biochemical analysis, satisfaction questionnaire | Nursing staff | The intervention can decrease the occurrence of inflammatory reaction, enhance nursing satisfaction, and enhance the children recovery process (total effective rate (%): case= 94.54, control= 77.78; nursing satisfaction (%): case= 96.36, control= 84.44; complication rate (%): case= 5.45, control=46.67) | Q3 |
| Wenxia Luo (2024) (18) | China | Case-control | 140 (Case= 70, control= 70) | Male-female | Case= 44.00 months, control= 40.50 months | Severe pneumonia | Bundled care combined with animated  educational videos (environmental care, providing a  warm ward, posting cute cartoon characters in the room,  placing some animated books and small toys, respiratory care, providing oxygen inhalation, administering nebulization inhalation, administering back tapping, oral care, dietary care ) | - | X-ray examination | Nursing staff | The intervention can enhance the children recovery process (body temperature recovery time: case= 7.08, control= 10.2; Recovery time of blood oxygen saturation: case= 6.43, control= 8.03; respiratory rate recovery time: case= 5.86, control= 7.87) | Q2 |
| Feyzullah Cetinkaya (2004) (19) | Turkey | Cross-sectional | 97 (group A: penicillin  G + chloramphenicol=46, group B: ceftriaxone= 51) | Male-female | 2-24 months | Severe pneumonia | Medication program (penicillin G + chloramphenicol versus ceftriaxone) | 10 days | Chest film examination, pulse oximeter | Nurse | Penicillin G+ chloramphenicol intervention may be a cheaper alternative to ceftriaxone intervention | Q1 |
| Tao Yu (2024) (20) | China | Case-control | 80 (Case= 40, control= 40) | Male-female | Case= 6.52, control= 6.63 | Severe pneumonia | Targeted sedation nursing combined with comprehensive nursing (maintaining comfortable temperature and humidity, providing quiet and tidy room, pulmonary physiotherapy, oral and nasal cavity care, establishing a targeted sedation team, setting sedation goals, administration of Dexmedetomidine  sedation, sedation assessment, monitoring of vital signs) | 6 months | Ramsay sedation scale (RSS), visual analogue scale (VAS), anxiety self-assessment scale (SAS), self-rating depression scale (SDS), social support scale | Nurse, doctor | The intervention can effectively decrease the occurrence of adverse reactions in the patients,  decrease the pain and discomfort of the patients, and significantly enhance the degree of sedation (RSS (mean): case= 2.5, control= 8.6; SAS (mean): case= 51.4, control= 55.8; SDS (mean): case= 44.2, control= 51.3; social support score (mean): case= 70.3, control= 59.7) | Q2 |
| Qiuying Xiao (2022) (21) | China | Case-control | 70 (Case= 35, control= 35) | Male-female | Case= 6.89, control= 6.10 | Mycoplasma pneumoniae pneumonia | Comfortable nursing based on optimized mobile internet of things (MIoT) (sedation based on MIoT, maintaining comfortable temperature and humidity, providing quiet and tidy room, performing oxygen inhalation, pulmonary care education ) | - | CT examination, pulmonary function test, Connor-Davidson resilience scale (CD-RISC), Frankl scale, general comfort questionnaire  (GCQ) | Nursing staff | The intervention can more successfully relieve the emotions of the patients (comfort score (mean): case= 95.01, control= 55.23; FEV1 (%): case= 68.26, control= 85.71; FEV1/FVC (%): case= 64.38, control= 74.22; incidence rate (%): case= 14.0, control= 46.0) | Q3 |
| Lieselotte Corten (2017) (22) | South Africa | Case-control | 29 (Case= 14, control= 15) | Male-female | Case= 7.7 months, control= 3.1 months | Uncomplicated pneumonia | Assisted autogenic drainage (AAD) | 5 days | Medical records, lung function test, oxygen saturation test | Physiotherapist | The intervention was effective (hospitalization (median): case= 7.0, control= 7.5) | Q2 |
| Shali Wu (2025) (23) | China | Case-control | 255 (Case= 131, control= 124) | Male-female | Case= 57.9 months, control= 54.2 months | Severe adenoviral pneumonia | Evidence-based nursing (airway assessment, positioning care, airway humidification, oral care, suctioning care, infection prevention and control, psychological support) | - | Medical records, blood biochemical analysis | Nurse | The intervention can enhance outcomes for the patients (overall efficiency (%): case= 98.47, control= 95.97; complication rate (%): case= 9.16, control= 25.0) | Q2 |
| Hao Zhang (2025) (24) | China | Case report | 1 | Female | 11 days | Severe pneumonia | Perioperative nursing care program (monitoring vital signs, respiratory care, preoperative bowel preparation, nutritional support, temperature management, liquid pathway management, position management, medication care, wound care) | - | Physical examination, laboratory tests, ultrasound imaging | Nursing staff | The intervention enhanced outcomes for the patient | Q1 |
| Yuxiao Hu (2025) (25) | China | Case-control | 50 (Case= 25, control= 25) | Male-female | Case= 6.0, control= 6.0 | Severe pneumonia | Integrated nursing care program (medication management, respiratory support, monitoring of vital signs, environmental care, nutrition support, establishing a multidisciplinary team, individualized treatment plan, respiratory rehabilitation training, continuous monitoring, parent skill training, family nursing kit, personalized supervision) | - | Lung function tests, laboratory inflammatory tests, nursing satisfaction questionnaire | Nurse, physician, rehabilitation  therapist, nutritionist, pulmonologist | The intervention enhanced outcomes for the patient (overall effectiveness (%): case= 100, control= 84.0; nursing satisfaction (%): case= 96.0, control= 76.0; complication rate (%): case= 8.0, control= 20.0; FEV1/FVC (%): case= 90.51, control= 88.72; hospital stays (d): case= 9.68, control= 12.16) | Q1 |
| Lei Ye (2023) (26) | China | Case-control | 230 (Case= 110, control= 120) | Male-female | - | Severe pneumonia | Pediatric early warning score combined with situation-background-assessment recommendation program | 6 months | Neonatal infant pain scale (NIPS), neonatal behavior neurological assessment scale (NBNA), nurse–physician questionnaire | Nurse, physician | The intervention was effective (incidence of handover problems (%): case= 9.09, control= 19.17) | Q2 |
| Jinying Lu (2024) (27) | China | Case-control | 80 (Case= 40, control= 40) | Male-female | Case= 4.24, control= 4.77 | Pneumonia | Hierarchical chain nursing care program (positioning care, dietary care, and monitoring of vital signs) | - | Lung function tests, self-rating anxiety scale (SAS), self-rating depression scale (SDS), nursing satisfaction questionnaire | Nursing staff | The intervention shows to be significantly effective in managing pneumonia in the patients (total effective rate (%): case= 97.5, control= 77.5; nursing satisfaction (%): case= 97.5, control= 75.0; hospital stay: case= 6.85, control= 9.83; PEF (ml/s): case= 66.78, control= 59.28; FRC (ml): case= 91.32, control= 82.68; ; FEF25% ml/s): case= 54.69, control= 49.98; SAS (mean): case= 47.73, control= 54.37; SDS (mean): case= 42.62, control= 50.07) | Q3 |
| Xiaoyan Liu (2025) (28) | China | Case-control | 90 (Case= 45, control= 45) | Male-female | Case= 11.82, control= 12.02 | Severe pneumonia | Comprehensive nursing based on the 3H model (monitoring of vital signs, respiratory care, psychological care, ward optimization, hotel-style (hotel) service, hospital-style (hospital) service, home-style (home) service) | - | Blood gas analysis, lung function tests, nursing satisfaction questionnaire, self-designed children’s treatment compliance scale | Nursing staff | The intervention shows to be significantly effective in managing pneumonia in the patients (length of hospital stay (mean): case= 8.56, control= 15.16; FEV1 (L): case= 3.54, control= 2.81; FVC (L): case= 2.34, control= 1.76; MVV (L): case= 59.47, control= 51.75; PEF (L/min): case= 198.64, control= 178.32; FEV1/FVC: case= 80.57, control= 72.71; nursing satisfaction (%): case= 97.78, control= 82.22; compliance rate (%): case= 75.56, control= 93.33) | Q3 |
| Novita Agustina (2021) (29) | Indonesia | Case-control | 32 (Case= 16, control= 16) | Male-female | 10.38 months | Pneumonia | Position care program (right lateral position) | - | Observation sheet | Nursing staff | The intervention improved oxygen saturation and respiratory rate in the patients (oxygen saturation: case= 98.50, control= 96.44; case= 43.38, control= 55.69) | Q3 |
| Aihua Qin (2023) (30) | China | Case-control | 200 (Case= 100, control= 100) | Male-female | Case= 5.32, control= 5.57 | Pneumonia | Intelligent nursing based on  cloud computing of internet of things (wireless temperature monitoring care, vital signs monitoring care, exchanging information between nurses and parents via wireless network) | - | Serological tests, pulmonary function tests, immune function tests | Nurse | The intervention shows to be significantly effective in managing pneumonia in the patients (FEV1 (l): case= 1.74, control= 1.64; FVC (l): case= 2.31, control= 2.23; PEF (l/s): case= 3.36, control= 3.23; vomiting (case): case= 2, control= 11; dry mouth (case): case= 3, control= 11; pruritus (case): case= 2, control= 9) | Q3 |
| Ruixue Sun (2025) (31) | China | Case-control | 160 (Case= 80, control= 80) | Male-female | Case= 8.30, control= 8.59 | Mycoplasma pneumonia | Targeted nursing care program (targeted psychological care, targeted dietary care, targeted medication care, preoperative and postoperative care) | - | Comfort-behavior scale, Frankl compliance scale, screen for child anxiety related emotional disorders (SCARED), depression self-  rating scale for children (DSRSC), positive and negative affect  scale (PANAS), Risser  patient satisfaction scale | Nursing staff | The intervention was significantly effective (comfort levels (mean): case= 7.14, control= 10.53; compliance (%): case= 95.0, control= 83.75; SCARED score (mean): case= 35.68, control= 42.49; DSRSC score (mean): case= 12.59, control= 17.84; positive emotion (mean): case= 34.08, control= 29.16; negative emotion (mean): case= 16.33, control= 22.30; nursing satisfaction (mean): case= 93.99, control= 83.04) | Q1 |
| Yuan Yue (2023) (32) | China | Case-control | 80 (Case= 40, control= 40) | Male-female | Case= 6.32, control= 6.27 | Pneumonia | Comprehensive nursing program (environmental care, health guidance, oxygen inhalation therapy, psychological care, dietary care, exercise) | 6 months | Imaging examination, patient satisfaction questionnaire short form (PSQ-18) | Nursing staff | The intervention was significantly effective (total effective rate (%): case= 97.5, control= 82.5; nursing satisfaction (%): case= 100, control= 90; hospitalization  time: case= 8.23, control= 9.57) | Q1 |
| Meseret Gebre (2024) (33) | Ethiopia | Case-control | 1240 (Case= 620, control= 620) | Male-female | Case= 11.0 months, control= 10.0 months | Severe pneumonia | Application of bubble continuous positive airway pressure | - | Medical records | Nursing staff | The intervention decreased the risk of treatment failure and in-hospital mortality in the patients (Severe hypoxaemia (%): case= 0.3, control= 2.6; mechanical ventilation (%): case= 0, control= 0.2; death in the hospital (%): case= 0, control= 1.0) | Q1 |
| Wen Chun Lin (2021) (34) | China | Cross-sectional | 41 | Male-female | 20 months | Exogenous lipoid pneumonia | Perioperative care program (preoperative examinations, explanation of the process for parents, psychological care, providing a warm and friendly environment, preoperative preparation, operative care, postoperative rehabilitation, health education, discharge guidance) | - | FLACC scale | Nurse | The intervention was effective | Q1 |
| Ying Shi (2020) (35) | China | Case-control | 106 (Case= 53, control= 53) | Male-female | Case= 2.41, control= 2.27 | Severe pneumonia | Quantitative assessment-based nursing program (providing a clean and tidy ward, environmental care, anti-infective treatment, rehabilitation care, health education, psychological education) | - | X-ray film examination, pulmonary function tests, nursing satisfaction questionnaire | Nursing staff | The intervention shows to be significantly effective in managing pneumonia in the patients (infection rate (%): case= 7.55, control= 22.64; VC (mL): case= 76.93, control= 63.29; MVV (mL): case= 71.31, control= 62.27; FEV1%: case= 68.02, control= 59.26; time to the disappearance of lung rale (d): case= 5.62, control= 11.37; length of hospital stay (d): case= 8.01, control= 10.81; complication rate (%): case= 5.66, control= 20.75) | Q3 |
| Xiaofei Xie (2025) (36) | China | Case-control | 92 (Case= 46, control= 46) | Male-female | Case= 8.0, control= 8.0 | Mycoplasma pneumoniae pneumonia | Qingke Mixture with  azithromycin | 6 days | Standardized clinical evaluation checklist, X-ray examination, laboratory inflammatory tests, pulmonary function tests, biochemical tests | Nurse, physician | The intervention significantly enhances outcomes in the patients (total effective rate (%): case= 95.65, control= 80.43; complication rate (%): case= 6.52, control= 10.87; FEV1 (%): case= 1.57, control= 1.32; FVC (%): case= 1.74, control= 1.66; FEV1/FVC (%): case= 0.89, control= 0.86) | Q1 |
| Xiuxiu Zhu (2025) (37) | China | Case-control | 98 (Case= 49, control= 49) | Male-female | Case= 2.35, control= 2.29 | Severe pneumonia | Family-centered nursing care program (team establishment,  Physical examination, Individualized family guidance, oral care, respiratory care, health education, online support, discharge guidance) | 2 weeks | Blood gas analyzer, schedule for the assessment of insight (SAI), short form-36 health status questionnaire (SF-36), client satisfactionquestionnaire-8 (CSQ-8), measure of processes of care 20-item (MPOC-20 | Nurse, pediatrician | The intervention significantly enhances outcomes in the patients (length of ICU stay (d): case= 6.12, control= 8.69; Psychological emotion (mean): case= 5.23, control= 4.68; Physical function (mean): case= 76.12, control= 67.09; Social function (mean): case= 79.25, control= 70.37; Emotional function (mean): case= 75.17, control= 65.3; MPOC-20 score (mean): case= 5.53, control= 4.68; (CSQ-8 score (mean): case= 30.11, control= 27.3) | Q3 |
| Yuyan Bi (2021) (38) | China | Case-control | 98 (Case= 49, control= 49) | Male-female | - | Mycoplasma pneumonia | Specific nursing care program (physical  sign nursing, psychological care, nutrition care, environmental care, education) | 15 days | CT examination, nursing satisfaction questionnaire | Nursing staff | The intervention significantly enhances outcomes in the patients (nursing satisfaction (%): case= 97.0, control= 88) | Q1 |
| Lubaba Shahrin (2021) (39) | Bangladesh | Cross-sectional | 308 (group A: amoxicillin group= 154, group B: ampicillin group= 154) | Male-female | Group A= 7.2 months, group B= 7.05 months | Severe pneumonia | Medication program (administration of amoxicillin and gentamicin) | 7 days | Pulse oximeter, blood analysis, X-ray examination | Nursing staff | It was not observed any difference between 2 interventions | Q1 |
| Nagwa A Zain ELdin (2018) (40) | Egypt | Cross-sectional | 40 | - | <6 months-10 years | Pneumonia | Nursing care protocol | 15 days | Observational checklist | Nursing staff | The intervention reduced the period of hospitalization of the patients (period of hospitalization: pre-intervention= 3.67, post-intervention= 1.90) | Q1 |

**References**

1.	Fu M, Lu H, Fu J, Sun H. Effects of comfortable nursing combined with video education on aerosol inhalation compliance and psychological state of children with bronchopneumonia. Archives of Clinical Psychiatry. 2022;49(5).

2.	Du M, Jia H, Zhao T, Liu Y, Wang D, Wang W. The clinical value of serum sST2 and cfDNA in guiding evidence-based nursing care for children with severe pneumonia complicated by myocardial damage. Journal of Medical Biochemistry. 2025;44(4):748-58.

3.	Liu Y, Ren H, Guo J, Su D. Effect of continuous nursing on nursing quality and patient quality of life and satisfaction among children with pneumonia. Journal of International Medical Research. 2021;49(3):0300060521993691.

4.	Fu S, Sun C, Shang X, Liu X. High quality nursing of children with pneumonia complicated with heart failure. Journal of biological regulators and homeostatic agents. 2019;33(3):905-10.

5.	Hua Z, Shu L, Wu X, Zhu Q, Li C. Combination of Qingfei Huayu Decoction and Targeted Nursing Care Improves the Clinical Outcomes in Pediatric Patients with Mycoplasma Pneumoniae Pneumonia. Journal of Biobased Materials and Bioenergy. 2024;18(2):281-7.

6.	Wang H. Analysis of ultrasonic atomized inhalation of antibiotics in infant pneumonia treatment. Pakistan Journal of Pharmaceutical Sciences. 2018;31(4 SI):1653-8.

7.	Wang M, Wu J. The Effect of the Hierarchical Chain Nursing Model Combined with Feilike Mixture Treatment on the Disappearance of Fever and Cough in Pediatric Pneumonia. Journal of Biobased Materials and Bioenergy. 2023;17(5):616-23.

8.	Rosuliana NE, Mawaddah E, Nurhayati T, Ningsih MU, Biswas HB. The Effect of a Continuous Nursing Care Program on the Quality of Life of Toddlers with Pneumonia, Parental Stress Levels, and Parental Satisfaction. Jurnal Kesehatan Prima. 2025;19(1):18-27.

9.	Cai J, Wu Q, Zhao W, Wang X, Lu M, Xu Q. Clinical impact of bronchoscopy combined with targeted nursing of oxygen nebulization inhalation in pediatric mycoplasma lobar pneumonia. African Journal of Reproductive Health/La Revue Africaine de la Santé Reproductive. 2025;29(5s):105-11.

10.	Zhang M. Improvement of IL-4, IL-6, IL-10, TNF-α and IFN-γ in children with mycoplasma pneumonia through the combination of video scenario-based breathing training and antibiotics. Sleep and Breathing. 2025;29(1):76.

11.	Huang D, Li R, Chen Y, Li X, Zhan Z, Li X, et al. Nursing care of a child with severe interstitial pneumonia complicated by acute rejection after lung transplantation: a case report. Translational Pediatrics. 2023;12(4):787.

12.	Onono M, Abdi M, Mutai K, Asadhi E, Nyamai R, Okoth P, et al. Community case management of lower chest indrawing pneumonia with oral amoxicillin in children in Kenya. Acta Paediatrica. 2018;107:44-52.

13.	Parvez M, Wiroonpanich W, Naphapunsakul M. The effects of educational program on child care knowledge and behaviors of mothers of children under five years with pneumonia. Bangladesh Journal of Medical Science. 2010;9(3):136-42.

14.	Chen J, Pan L, Qiu Y, Jin L. Efficacy of the PDCA cyclic care model in improving lung function and speed of recovery in children with mycoplasma pneumonia. American Journal of Translational Research. 2025;17(3):1792.

15.	Keeley D, Nkrumah F, Kapuyanyika C. Randomized trial of sulfamethoxazole+ trimethoprim versus procaine penicillin for the outpatient treatment of childhood pneumonia in Zimbabwe. Bulletin of the World Health Organization. 1990;68(2):185.

16.	Yang F. Oxygen-driving and atomized mucosolvan inhalation combined with holistic nursing in the treatment of children severe bronchial pneumonia. Pakistan Journal of Pharmaceutical Sciences. 2015;28(4):1477-81.

17.	ZHANG Y, LI A. The effect of risk preventive nursing on clinical curative effect and rehabilitation in children with pneumonia. Chinese Journal of Integrated Traditional and Western Medicine in Intensive and Critical Care. 2019:350-3.

18.	Luo W, Liu Y, Wen C, Chen X, Zhang J. Bundled care combined with animated educational videos can promote the recovery of severe pneumonia in children: A case-control study. Medicine. 2024;103(22):e38437.

19.	Cetinkaya F, Gogremis A, Kutluk G. Comparison of two antibiotic regimens in the empirical treatment of severe childhood pneumonia. The Indian Journal of Pediatrics. 2004;71(11):969-72.

20.	Yu T, Wang N, Li A, Xu Y. Clinical evaluation of targeted sedation nursing combined with comprehensive nursing in children with severe pneumonia. Medicine. 2024;103(1):e36317.

21.	Xiao Q, Wu B, Wu W, Wang R. Adoption and safety evaluation of comfortable nursing by mobile internet of things in pediatric outpatient sedation. Computational and Mathematical Methods in Medicine. 2022;2022(1):3257101.

22.	Corten L, Jelsma J, Human A, Rahim S, Morrow BM. Assisted autogenic drainage in infants and young children hospitalized with uncomplicated pneumonia, a pilot study. Physiotherapy Research International. 2018;23(1):e1690.

23.	Wu S, Zhu S, Wen H, Yang T, Liu Y, Peng Y. Evaluating the effects of evidence-based nursing on length of hospital stay, duration of mechanical ventilation, symptom relief, and complication rates in children with severe adenoviral pneumonia: a prospective randomized controlled trial. Revista do Instituto de Medicina Tropical de São Paulo. 2025;67:e13.

24.	Zhang H, Zhang M. Perioperative management of a neonate with congenital biliary atresia complicated by severe pneumonia undergoing hepatic hilar jejunostomy: A case report. Nursing in Critical Care. 2025.

25.	Hu Y, Guo Q, Liu X, Lv W, Liu L. Integrated nursing and medical management improves outcomes in pediatric lobar pneumonia: a randomized controlled study. Frontiers in Pediatrics. 2025;13:1612618.

26.	Ye L, Zhu M, Hong F, Zhang W, Song L. The value of Pediatric Early Warning Score combined with SBAR in neonatal pneumonia nursing: A retrospective cohort study. Medicine. 2023;102(10):e33197.

27.	Hao M, Lu J, Yin Z, Chang T, Liu L. The Impact of Hierarchical Chain Nursing Model on the Resolution Time of Fever and Cough in Children with Pneumonia. J Mod Nurs Pract Res. 2024;4.

28.	Liu X, Jin J, Lin C, Ye W, Xu X, Chen X. Impact of comprehensive nursing based on the" 3H" theory on blood gas, recovery speed and lung function in children with severe pneumonia. Signa Vitae. 2025;21(4).

29.	Agustina N. Right lateral position can improving oxygen saturation and respiratory rate on under-five children with pneumonia. 2021.

30.	Qin A, Liu Y, Shao C, Dong H. Application of intelligent nursing based on cloud computing of internet of things in children with pneumonia and sepsis treated with human gamma globulin. European Journal of Inflammation. 2023;21:1721727X231194144.

31.	Sun R, Zhang Y, Cheng F, Zhao Y. Effect of pulmonary rehabilitation nursing approach with integrated traditional Chinese and western medicine in children with Mycoplasma Pneumonia after Bronchoalveolar Lavage. Pakistan Journal of Medical Sciences. 2025;41(4):1105.

32.	Yue Y, Liang Q, Shi L, Bai W-j, Fu J. Effect of comprehensive nursing intervention on the efficacy of spleen aminopeptide combined with aerosol inhalation in the treatment of pediatric pneumonia. Pakistan Journal of Medical Sciences. 2023;39(4):1086.

33.	Gebre M, Haile K, Duke T, Faruk MT, Kamal M, Kabir MF, et al. Effectiveness of bubble continuous positive airway pressure for treatment of children aged 1–59 months with severe pneumonia and hypoxaemia in Ethiopia: a pragmatic cluster-randomised controlled trial. The Lancet Global Health. 2024;12(5):e804-e14.

34.	Lin W-C, Qin Y, Chun X, Huang R-L, Chen R-S, Zhang D. Perioperative care of children with acute exogenous lipoid pneumonia submitted to BAL/FB. International Journal of General Medicine. 2021:8383-8.

35.	Shi Y, Zhang Q, Zhao C, Guo S, Ren L, Ma H, et al. The effect of quantitative assessment on the nursing of children with severe pneumonia. Int J Clin Exp Med. 2020;13(2):847-56.

36.	Xie X, Wang W, Cui S, Xie T, Song Y, Wang N, et al. Clinical Efficacy of Qingke Mixture in Treating Mycoplasma Pneumonia in Children: A Randomized Controlled Trial. International Journal of General Medicine. 2025:5073-83.

37.	Zhu X, Yang J, Yang L, Su J. Effects of Family-Centered Nursing on Psychological Well-Being and Quality of Life in Children with Severe Pneumonia in the Intensive Care Unit: A Prospective Study. Iran J Pediatr. 2025;35(2):e146594.

38.	Bi Y, Jiang C, Qi H, Zhou H, Sun L. Computed tomography image texture under feature extraction algorithm in the diagnosis of effect of specific nursing intervention on mycoplasma pneumonia in children. Journal of Healthcare Engineering. 2021;2021(1):6059060.

39.	Shahrin L, Chisti MJ, Sarmin M, Rahman ASMMH, Shahid ASMSB, Islam MZ, et al. Intravenous amoxicillin plus intravenous gentamicin for children with severe pneumonia in Bangladesh: An open-label, randomized, non-inferiority controlled trial. Life. 2021;11(12):1299.

40.	Zain ELdin N, Omar T, Ahmed H, Kamar L. Effect of A Nursing Care protocol on the Duration of Hospitalization of Children with Pneumonia. Menoufia Nursing Journal. 2018;3(2):1-11.
